# Supplementary material for: Modeling glioblastoma heterogeneity as a dynamic network of cell states
Source: Mol Syst Biol. 2021 Sep 16;17(9):e10105. doi: 10.15252/msb.202010105 (PMC8444284; doi:10.15252/msb.202010105)
Supplement: Supplementary file 6 — Source Data for Figure 5 [file MSB-17-e10105-s004.zip › Figure5A_sourcedata/GSEA_3017/nosigdb_stateF.GseaPreranked.1622795015644/SUVA_G2M_PMID_31327527.html]

Details for gene set SUVA\_G2M\_PMID\_31327527[GSEA]

|  || Dataset | state33017 |
| Phenotype | NoPhenotypeAvailable |
| Upregulated in class | na\_neg |
| GeneSet | SUVA\_G2M\_PMID\_31327527 |
| Enrichment Score (ES) | -0.8508665 |
| Normalized Enrichment Score (NES) | -4.30053 |
| Nominal p-value | 0.0 |
| FDR q-value | 0.0 |
| FWER p-Value | 0.0 |
Table: GSEA Results Summary

  

Fig 1: Enrichment plot: SUVA\_G2M\_PMID\_31327527      
 Profile of the Running ES Score & Positions of GeneSet Members on the Rank Ordered List

  

| PROBE | GENE SYMBOL | GENE\_TITLE | RANK IN GENE LIST | RANK METRIC SCORE | RUNNING ES | CORE ENRICHMENT || 1 | OIP5 |  |  | 1686 | -0.314 | -0.7515 | No |
| 2 | KIF20A |  |  | 1792 | -0.358 | -0.7875 | No |
| 3 | MXD3 |  |  | 1905 | -0.428 | -0.8245 | No |
| 4 | MZT1 |  |  | 1936 | -0.447 | -0.8238 | No |
| 5 | SPAG5 |  |  | 1997 | -0.490 | -0.8352 | Yes |
| 6 | ARL6IP1 |  |  | 2001 | -0.494 | -0.8208 | Yes |
| 7 | RANGAP1 |  |  | 2011 | -0.502 | -0.8089 | Yes |
| 8 | CDCA3 |  |  | 2041 | -0.529 | -0.8051 | Yes |
| 9 | TROAP |  |  | 2046 | -0.533 | -0.7899 | Yes |
| 10 | TTK |  |  | 2047 | -0.533 | -0.7729 | Yes |
| 11 | RACGAP1 |  |  | 2072 | -0.579 | -0.7653 | Yes |
| 12 | KNSTRN |  |  | 2085 | -0.615 | -0.7511 | Yes |
| 13 | PLK1 |  |  | 2087 | -0.624 | -0.7316 | Yes |
| 14 | NCAPD2 |  |  | 2089 | -0.627 | -0.7121 | Yes |
| 15 | CKAP2L |  |  | 2109 | -0.663 | -0.6995 | Yes |
| 16 | AURKA |  |  | 2110 | -0.673 | -0.6781 | Yes |
| 17 | CDC20 |  |  | 2117 | -0.695 | -0.6586 | Yes |
| 18 | CDCA8 |  |  | 2129 | -0.722 | -0.6406 | Yes |
| 19 | CKAP2 |  |  | 2131 | -0.729 | -0.6178 | Yes |
| 20 | TUBA1C |  |  | 2135 | -0.756 | -0.5950 | Yes |
| 21 | KIF2C |  |  | 2136 | -0.756 | -0.5709 | Yes |
| 22 | KIFC1 |  |  | 2151 | -0.802 | -0.5516 | Yes |
| 23 | TUBB4B |  |  | 2162 | -0.843 | -0.5293 | Yes |
| 24 | ECT2 |  |  | 2163 | -0.846 | -0.5023 | Yes |
| 25 | HMGB3 |  |  | 2164 | -0.852 | -0.4751 | Yes |
| 26 | ARHGAP11A |  |  | 2167 | -0.855 | -0.4488 | Yes |
| 27 | BUB1 |  |  | 2170 | -0.860 | -0.4223 | Yes |
| 28 | FOXM1 |  |  | 2171 | -0.861 | -0.3948 | Yes |
| 29 | BUB1B |  |  | 2174 | -0.871 | -0.3679 | Yes |
| 30 | KPNA2 |  |  | 2178 | -0.894 | -0.3408 | Yes |
| 31 | CCNA2 |  |  | 2181 | -0.902 | -0.3129 | Yes |
| 32 | KIF4A |  |  | 2186 | -0.921 | -0.2854 | Yes |
| 33 | CCNB2 |  |  | 2200 | -0.983 | -0.2599 | Yes |
| 34 | KIF11 |  |  | 2206 | -1.008 | -0.2300 | Yes |
| 35 | H2AFZ |  |  | 2220 | -1.090 | -0.2011 | Yes |
| 36 | CCNB1 |  |  | 2224 | -1.148 | -0.1659 | Yes |
| 37 | ANP32E |  |  | 2227 | -1.200 | -0.1285 | Yes |
| 38 | SMC4 |  |  | 2236 | -1.281 | -0.0912 | Yes |
| 39 | TMPO |  |  | 2239 | -1.382 | -0.0481 | Yes |
| 40 | CENPF |  |  | 2249 | -1.691 | 0.0018 | Yes |
Table: GSEA details [plain text format]

  

Fig 2: SUVA\_G2M\_PMID\_31327527: Random ES distribution      
 Gene set null distribution of ES for **SUVA\_G2M\_PMID\_31327527**
